# Inhibiting the NLRP3 inflammasome with MCC950 ameliorates retinal neovascularization and leakage by reversing the IL-1*β*/IL-18 activation pattern in an oxygen-induced ischemic retinopathy mouse model

**DOI:** 10.1038/s41419-020-03076-7

**Published:** 2020-10-22

**Authors:** Ailing Sui, Xiuping Chen, Jikui Shen, Anna M. Demetriades, Yiyun Yao, Yixuan Yao, Yanji Zhu, Xi Shen, Bing Xie

**Affiliations:** 1 The Department of Ophthalmology, Ruijin Hospital, Shanghai Jiao Tong University School of Medicine, Shanghai, China; 2 The Department of Ophthalmology, Zhongshan Hospital, Fudan University, Shanghai, China; 3 The Departments of Ophthalmology and Neuroscience, The Johns Hopkins University School of Medicine, Baltimore, MA USA; 4 The Department of Ophthalmology, New York Presbyterian Hospital-Weill Cornell Medicine, New York, NY USA

**Keywords:** Drug discovery, Molecular biology, Cellular immunity, Inflammasome

## Abstract

Activation of the nucleotide-binding domain leucine-rich repeat and pyrin domain containing receptor 3 (NLRP3) inflammasome plays an important role in ocular neovascularization. In our study, we found that the expression and activation levels of NLRP3 inflammasome components, including NLRP3, an apoptosis-associated speck-like protein (ASC) containing caspase activation and recruitment domain (CARD) and caspase-1 (CAS1), were significantly upregulated. In addition, we found interleukin (IL)-1*β* activity increased while IL-18 activity decreased in the retinas of oxygen-induced ischemic retinopathy (OIR) mice. MCC950, an inhibitor of NLRP3, reversed the IL-1*β*/IL-18 activation pattern, inhibited the formation of retinal neovascularization (RNV), decreased the number of acellular capillaries and reduced leakage of retinal vessels. Moreover, MCC950 could regulate the expression of endothelial cell- and pericyte function-associated molecules, such as vascular endothelial growth factor (VEGF), VEGF receptor (VEGFR)1, VEGFR2, matrix metalloproteinase (MMP)2, MMP9, tissue inhibitor of metalloproteinases (TIMP)1, TIMP2, platelet-derived growth factor receptor-*β* (PDGFR-*β*), platelet-derived growth factor-B (PDGF-B), and angiopoietin2 (Ang2). In vitro, recombinant human (r)IL-18 and rIL-1*β* regulated the expression of endothelial cell- and pericyte function-associated molecules and the proliferation and migration of endothelial cells and pericytes. We therefore determined that inhibiting the NLRP3 inflammasome with MCC950 can regulate the function of endothelial cells and pericytes by reversing the IL-1*β*/IL-18 activation pattern to ameliorate RNV and leakage; thereby opening new avenues to treat RNV-associated ocular diseases.

## Introduction

Retinal neovascularization (RNV)-associated diseases such as retinal vein occlusion, diabetic retinopathy (DR), and retinopathy of prematurity (ROP), are characterized by pathological RNV and abnormal vascular permeability leading to retinal edema, hemorrhage, detachment, hyperplasia and other conditions, which seriously threaten vision and lead to visual decline^1–3^. Currently, intravitreal injection of anti-vascular endothelial growth factor (VEGF) is widely used in the treatment of RNV-associated diseases, but can only temporarily inhibit the formation of RNV. Moreover, repeated intravitreal administration is not only expensive but also prone to complications such as retinal detachment, cataracts, and endophthalmitis^4–6^. It is therefore important to further determine the etiology and pathological mechanism of RNV to find alternative or adjuvant therapies.

In recent years, researchers have highlighted the importance of immune-mediated inflammation in the occurrence, development and treatment of ocular NV diseases^7–10^. As a member of the nucleotide-binding and oligomerization domain (NOD)-like receptor family that induces the adaptive immune response, the nucleotide-binding domain leucine-rich repeat and pyrin domain containing receptor 3 (NLRP3) inflammasome is an important part of the aseptic inflammatory reaction and the core component of the inflammatory protein complex composed of the innate immune receptor NLRP3, apoptosis-associated speck-like protein (ASC) containing caspase activation and recruitment domain and pro-caspase-1 (pro-CAS1)^11,12^. When cells are stimulated by endogenous or exogenous factors, the expression of interleukin-1*β* (IL-1*β*) and IL-18 changes, and the NLRP3 inflammasome is activated at the same time. Activated CAS1 cleaves the precursors of IL-1*β* and IL-18 to generate activated IL-1*β* and IL-18, which are released from cells, thus triggering a series of inflammatory reactions^11^. In studies involving age-related macular degeneration (AMD), DR, and other retinopathies, researchers found that the NLRP3 inflammasome activation was the key factor leading to the proinflammatory effect, but the specific mechanism remains unknown^13,14^.

Given the existing literature regarding the NLRP3 inflammasome, we hypothesized that the NLRP3 inflammasome may be involved in the regulation of RNV. We therefore established an experimental RNV mouse model and performed in vitro cell studies to explore the role of the NLRP3 inflammasome and the possible mechanisms involved to provide a comprehensive experimental basis for the identification of new therapeutic targets for RNV-associated diseases.

## Results

### NLRP3 inflammasome expression and activation were significantly increased in the retinas of OIR mice

**Fig. 1:**
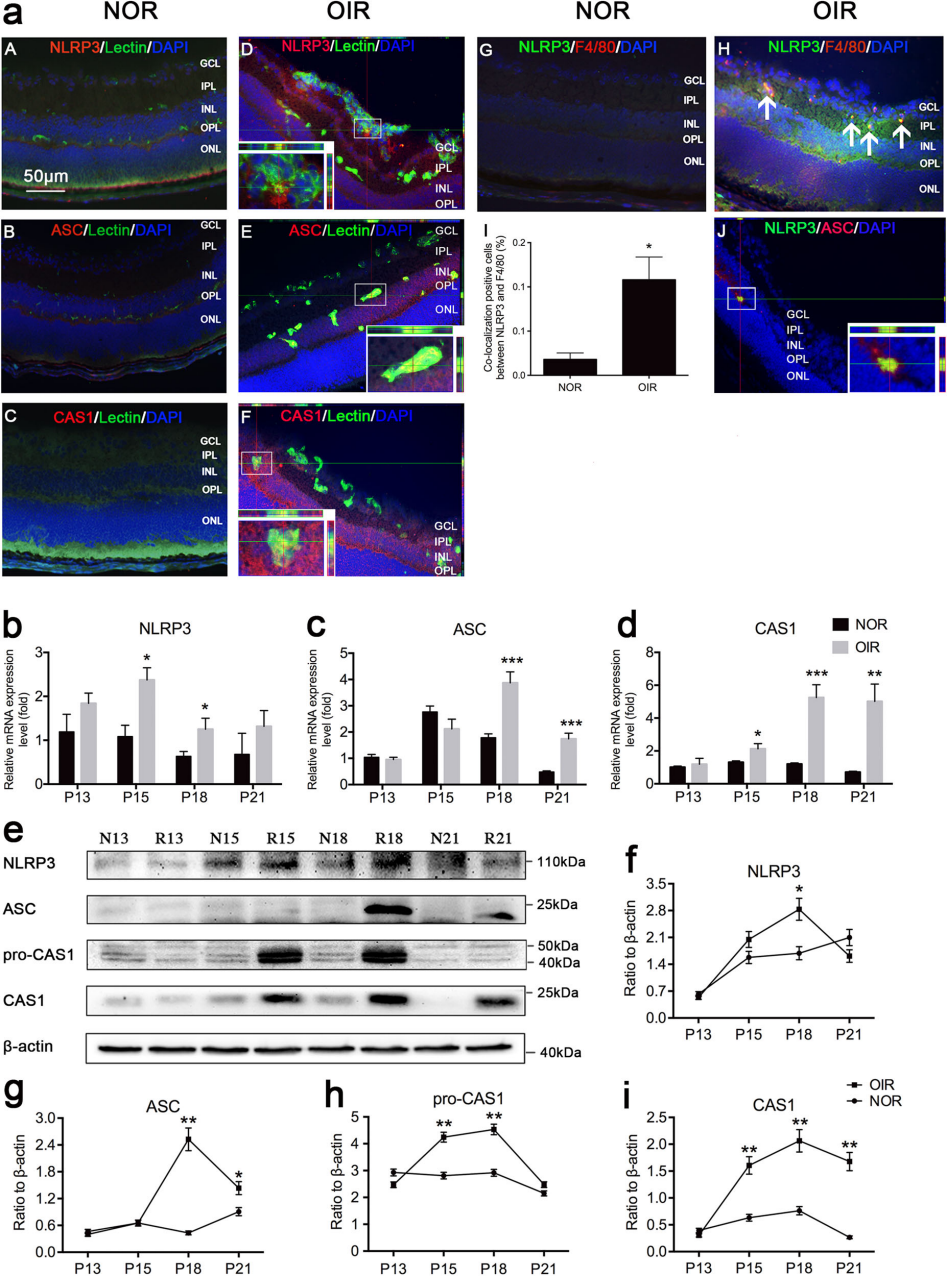
NLRP3 inflammasome expression and activity were significantly increased in the retinas of OIR mice. **a** Immunofluorescence staining for lectin (a marker of neovascularization) and NLRP3 (or ASC, CAS1), F4/80 (a marker of macrophages) and NLRP3, NLRP3 and ASC in the retinas of mice at P18 (*n* = 3 mice/group). The magnified pictures shown are representative colocalization images. Arrowheads point to positive areas. The colocalization of F4/80 and NLRP3 in the retinas of normal and OIR mice (*n* = 4 eyes /group) was quantified (aG–I). **b**–**d** qRT-PCR was performed to quantify the mRNA expression of NLRP3, ASC, and CAS1 at P13, P15, P18, and P21 (*n* = 6–8 mice/group). **e**–**i** Western blot analysis of the protein expression of NLRP3, ASC, and CAS1 (*n* = 3 mice/group). Data are presented as the mean ± SEM from three independent experiments and were analyzed by two-tailed Student’s *t* test. *P < 0.05, **P < 0.01, ***P < 0.001. The layers of the retina in cross-sections are shown: ganglion cell layer (GCL), inner plexiform layer (IPL), inner nuclear layer (INL), outer plexiform layer (OPL), and outer nuclear layer (ONL). NOR normal mice, OIR oxygen-induced ischemic retinopathy mice, P postnatal day, N13 normal mice at postnatal day 13, R13 oxygen-induced ischemic retinopathy mice at postnatal day 13, CAS1 caspase-1.

Quantitative reverse transcription polymerase chain reaction (qRT-PCR) results revealed that NLRP3, ASC, and CAS1 mRNA expression was upregulated in the retinas of OIR mice from P13 to P21 (Fig. 1b–d). In addition, western blot assays showed gradual overexpression and activation of the NLRP3 inflammasome (Fig. 1e–i). Immunofluorescent staining of normal and OIR mice indicated that high expression levels of NLRP3, ASC, and CAS1 in the inner layer of the retina of OIR mice compared with normal control. Merged images showed a small amount of colocalization between NLRP3 (or CAS1) and lectin, which stained endothelial cells, and more colocalization between NLRP3 (or CAS1) and F4/80, which stained macrophages (Fig. 1aA–H and Supplementary Fig. S1). This indicated that the NLRP3 inflammasome was expressed in both endothelial cells and macrophages, but more so in the latter. The analysis results of the immunofluorescent staining have shown that the colocalization positive cells between NLRP3 and F4/80 significantly increased in the retinas of OIR mice (Fig. 1aI, *P* < 0.05). We also observed the colocalization of NLRP3 with ASC which means the formation of inflammasome complexes in the retinas of OIR mice. The protein expression of NLRP3 and CAS1 in THP-1 cells was also upregulated after hypoxia stimulation in vitro (Supplementary Fig. S3). The immunofluorescent staining of cells displayed increased positive staining of NLRP3 and CAS1 and the magnified z-stacks of confocal images clearly showed the formation of ASC specks and the colocalization of NLRP3 and ASC in the hypoxia-induced THP-1 cells (Supplementary Fig. S2), which were consistent with the experimental results in vivo. We therefore concluded that the NLRP3 inflammasome was significantly activated in the retinas of OIR mice and might play an important role in RNV development.

### The expression and activation levels of IL-1*β* were upregulated, while IL-18 levels were downregulated in the retinas of OIR mice

**Fig. 2:**
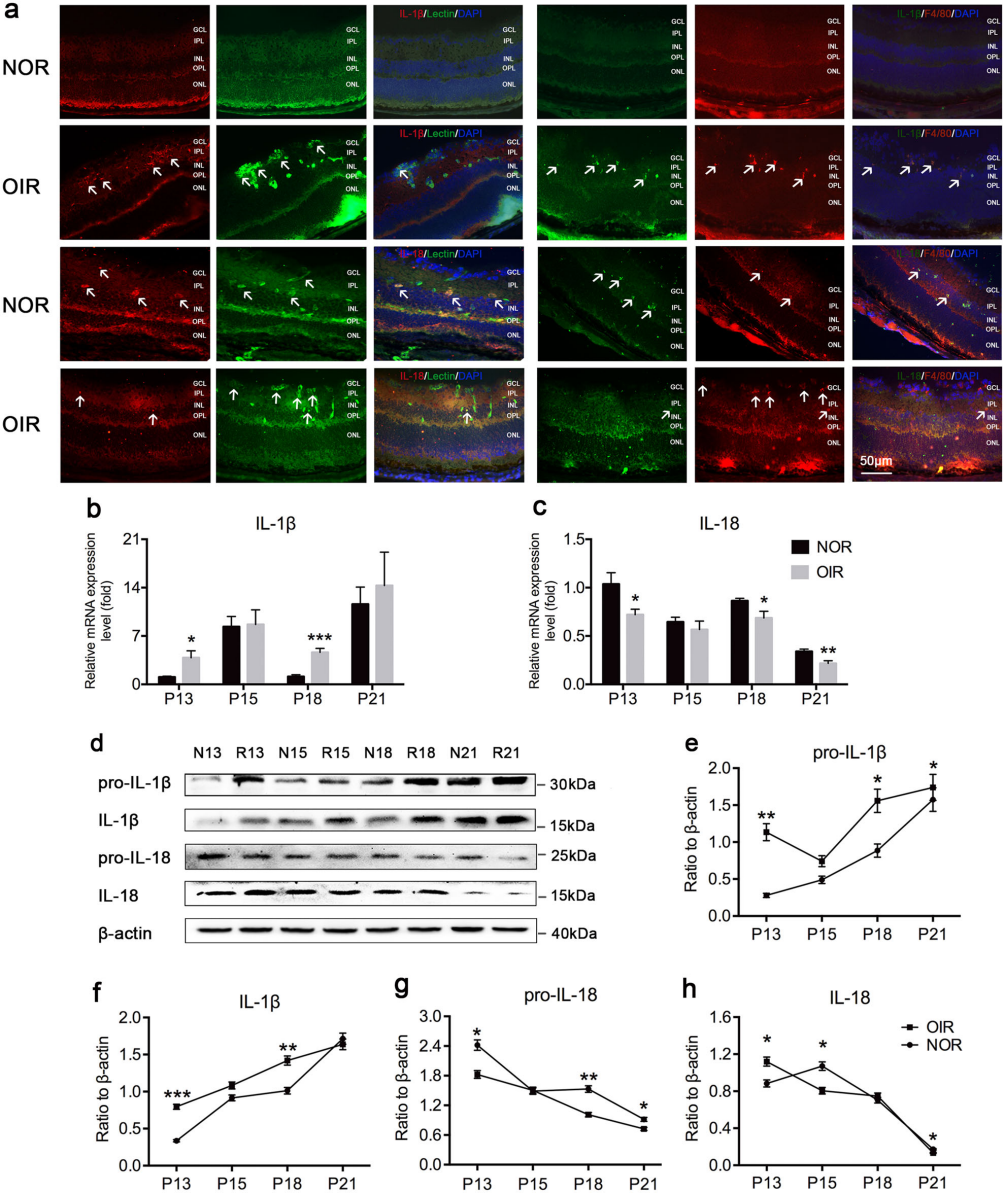
The expression and activation of IL-1*β* significantly increased while IL-18 significantly decreased in the retinas of OIR mice. **a** Immunofluorescence staining for lectin, F4/80, IL-1*β* and IL-18 in the retinas of mice at P18 (*n* = 3 mice/group). Arrowheads point to positive areas. The layers of the retina in cross-sections are shown: ganglion cell layer (GCL), inner plexiform layer (IPL), inner nuclear layer (INL), outer plexiform layer (OPL), and outer nuclear layer (ONL). **b**–**c** qRT-PCR was performed to quantify the mRNA expression of IL-1*β* and IL-18 at P13–P21 (*n* = 6–8 mice/group). **d**–**h** Western blotting was performed to detect the protein expression of pro-IL-1*β*, IL-1*β*, pro-IL-18, and IL-18 (*n* = 3 mice/group). Data are displayed as the mean ± SEM. Experiments were repeated three times independently. *P < 0.05, **P < 0.01, ***P < 0.001 calculated with two-tailed Student’s *t* test. NOR normal mice, OIR oxygen-induced ischemic retinopathy mice, P postnatal day, N13 normal mice at postnatal day 13, R13 oxygen-induced ischemic retinopathy mice at postnatal day 13.

The mRNA and protein expression results showed that the expression and activation of IL-1*β* increased, while the expression of IL-18 decreased, and the activation of IL-18 increased in the early stage of OIR (P13) and then decreased in the later stage (Fig. 2b–h). Immunofluorescence staining also showed that the expression of IL-1*β* increased, while the expression of IL-18 decreased in OIR mice. Merged images showed that both IL-1*β* and IL-18 had less colocalization with lectin and more colocalization with F4/80 (Fig. 2a). The IL-1*β* activation was upregulated while IL-18 activation was downregulated (Supplementary Fig. S3) and the immunofluorescent staining of cells also showed increased positive staining of IL-1*β* and decreased positive staining of IL-18 in THP-1 cells treated under hypoxic conditions (Supplementary Fig. S2), which were consistent with the experimental results in mice. All of these results suggest that IL-1*β* and IL-18 in the retinas of OIR mice have opposite activation patterns.

### MCC950 inhibited the activation of the NLRP3 inflammasome and reversed the activation pattern of IL-1*β*/IL-18

**Fig. 3:**
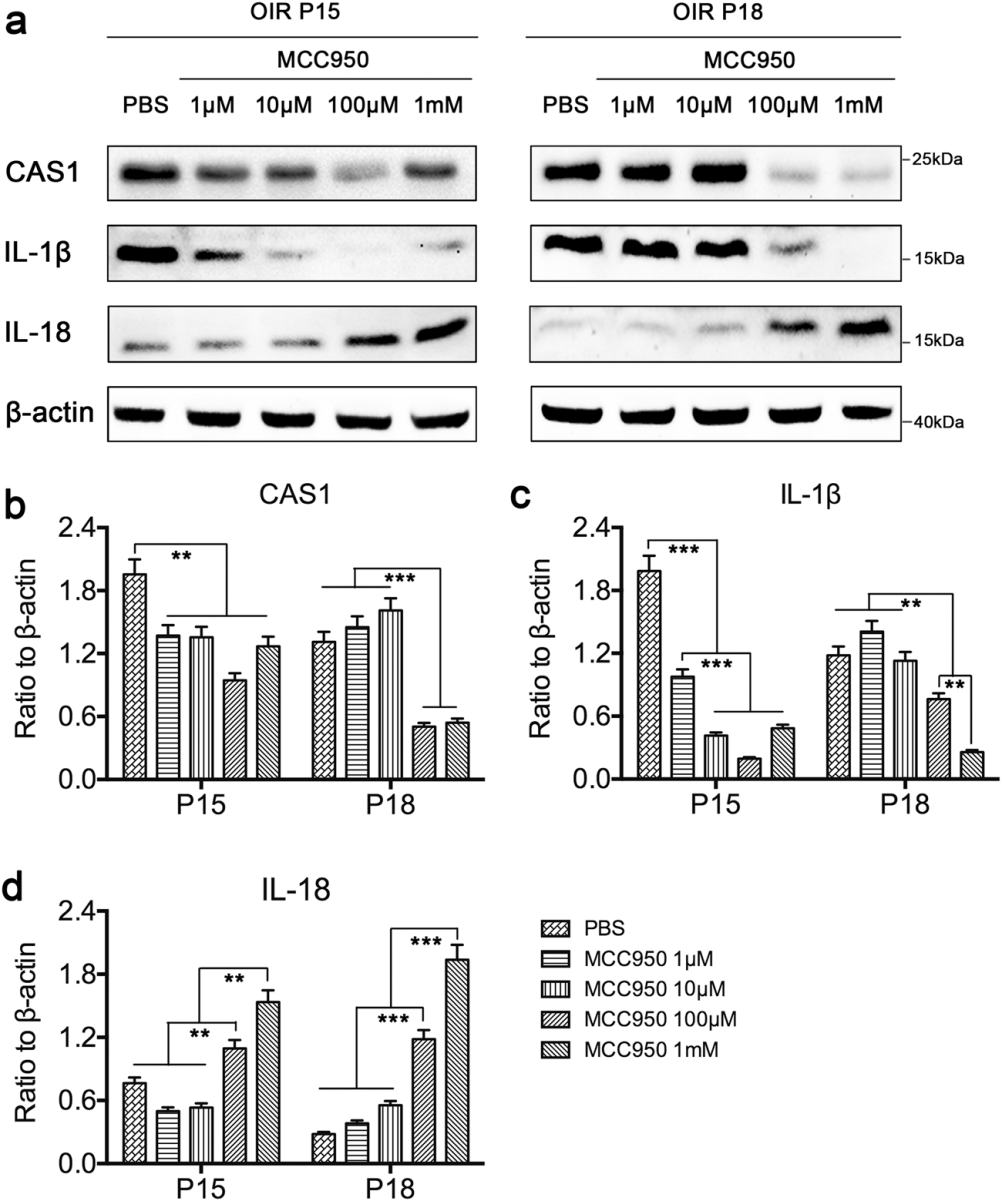
MCC950 inhibited the activation of the NLRP3 inflammasome and reversed the activation pattern of IL-1*β* and IL-18 in the retinas of OIR mice. **a** Western blot assay was used to detect the expression of CAS1, IL-1*β*, and IL-18 in the retinas of OIR mice intravitreally injected with MCC950 (1, 10, 100 μM, 1 mM) or PBS (*n* = 3 mice/group). **b**–**d** Quantification of the intensity of target protein bands normalized relative to *β*-actin band intensity. Data are presented as the mean ± SEM from three independent experiments and were analyzed by Student–Newman–Keuls (SNK). **P < 0.01, ***P < 0.001. OIR oxygen-induced ischemic retinopathy mice, P postnatal day.

The western blot results (Fig. 3) showed that MCC950 significantly inhibited CAS1 activation at concentrations of 100 μM and 1 mM. We also found a significant decrease in IL-1*β* activation and an increase in IL-18 activation in the OIR mice treated with MCC950 at concentrations of 100 μM and 1 mM. Similar results were obtained in vitro, i.e., MCC950 significantly inhibited the expression of NLRP3 and the activation of CAS1 and IL-1*β* but enhanced the activation level of IL-18 (Supplementary Fig. S4) in hypoxia-induced THP-1 cells. These results indicated that MCC950 inhibited the activation of the NLRP3 inflammasome and reversed the activation pattern of IL-1*β*/IL-18 in the retinas of OIR mice.

### MCC950 suppressed the development of RNV, decreased the number of acellular capillaries, and reduced the leakage of retinal vessels

**Fig. 4:**
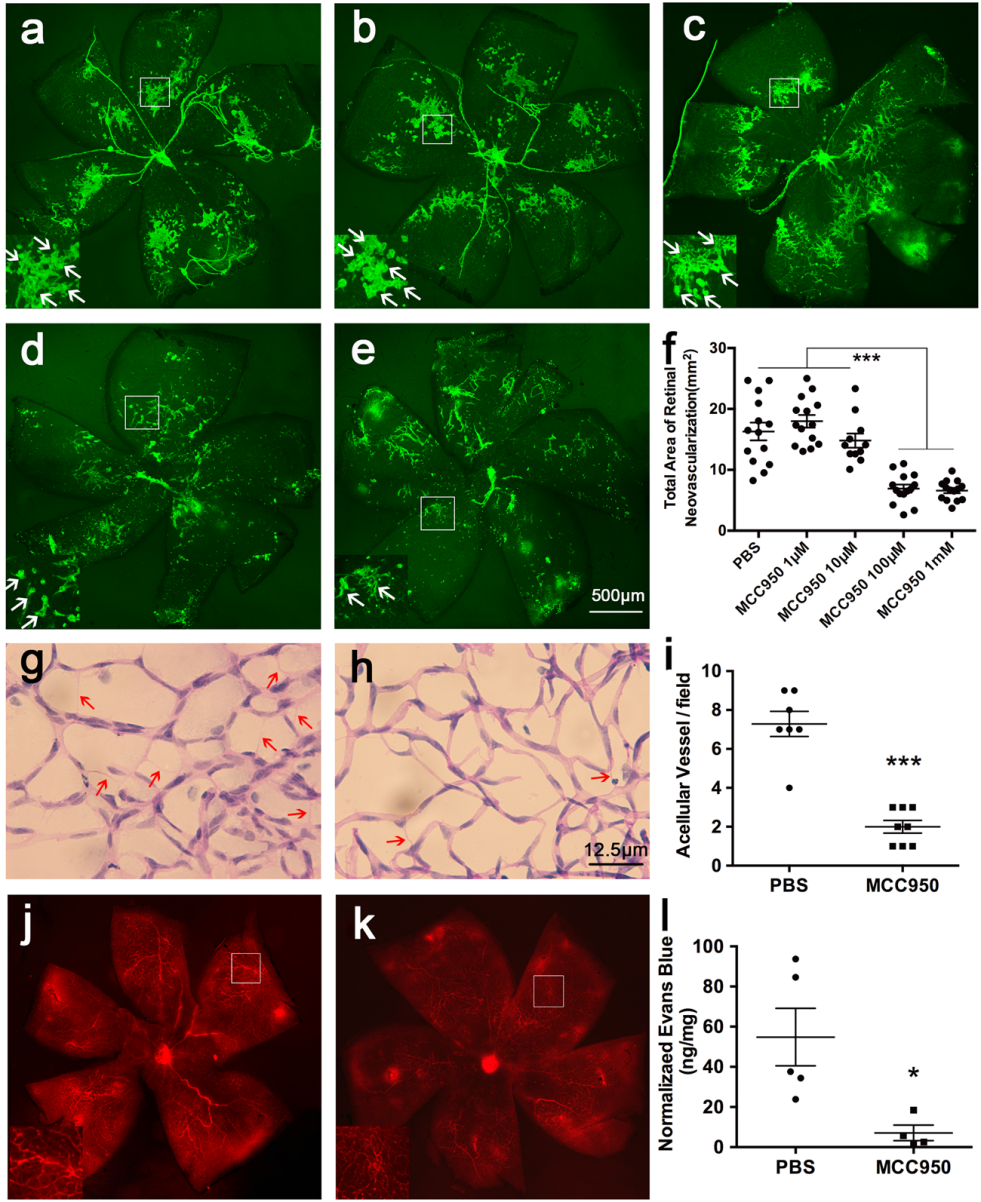
MCC950 suppressed the development of RNV, decreased the number of acellular capillaries and reduced the leakage of retinal vessels. One eye of OIR mice was injected with MCC950 at 1 μM (**b**, *n* = 14 eyes), 10 μM (**c**, *n* = 11 eyes), 100 μM (**d**, *n* = 14 eyes) or 1 mM (**e**, *n* = 14 eyes), and the contralateral eye was injected with PBS (**a**, *n* = 14 eyes) at P12. Retinas were stained with FITC-lectin and flat-mounted at P18. Arrowheads point to positive areas. Statistical analysis was performed using the SNK. ***P < 0.001. Retina trypsin digestion was performed to detect the number of acellular capillaries in PBS-treated (**g**, *n* = 3 eyes) and MCC950-treated (**h**, *n* = 3 eyes) mice. Acellular capillaries were quantified in 7–8 random fields (**i**) and marked with red arrows. ***P < 0.001. The OIR mice treated with PBS (**j**, *n* = 6 eyes) or MCC950 (**k**, *n* = 7 eyes) were infused with Evans blue dye and flat-mounted to detect the fluorescence signal. A representative image is shown above. **l** Quantification of Evans blue leakage was conducted in two groups: PBS group (*n* = 5 eyes) and MCC950 group (*n* = 4 eyes). Data are shown as the mean ± SEM and the statistical analyses were performed using two-tailed Student’s *t* test. *P < 0.05.

Retinal flat mounts showed that compared with the phosphate-buffered saline (PBS) group (Fig. 4a), the RNV areas were significantly reduced in the MCC950–100 μM and MCC950-1 mM groups (Fig. 4d, e, *P* < 0.001). After intravitreal injection of MCC950–100 μM, the number of acellular capillaries representing capillary degeneration were significantly reduced (Fig. 4g, h, *P* < 0.001). Evans blue assays showed that the level of retinal vascular leakage in the MCC950–100 μM group was significantly lower than that in the PBS group (Fig. 4j–l, *P* < 0.05). All these experimental results showed that inhibiting the activation of the NLRP3 inflammasome with MCC950 suppressed the development of RNV and reduced the leakage of retinal vessels.

### Intravitreal injection of MCC950 regulated the expression of several angiogenesis-associated molecules

**Fig. 5:**
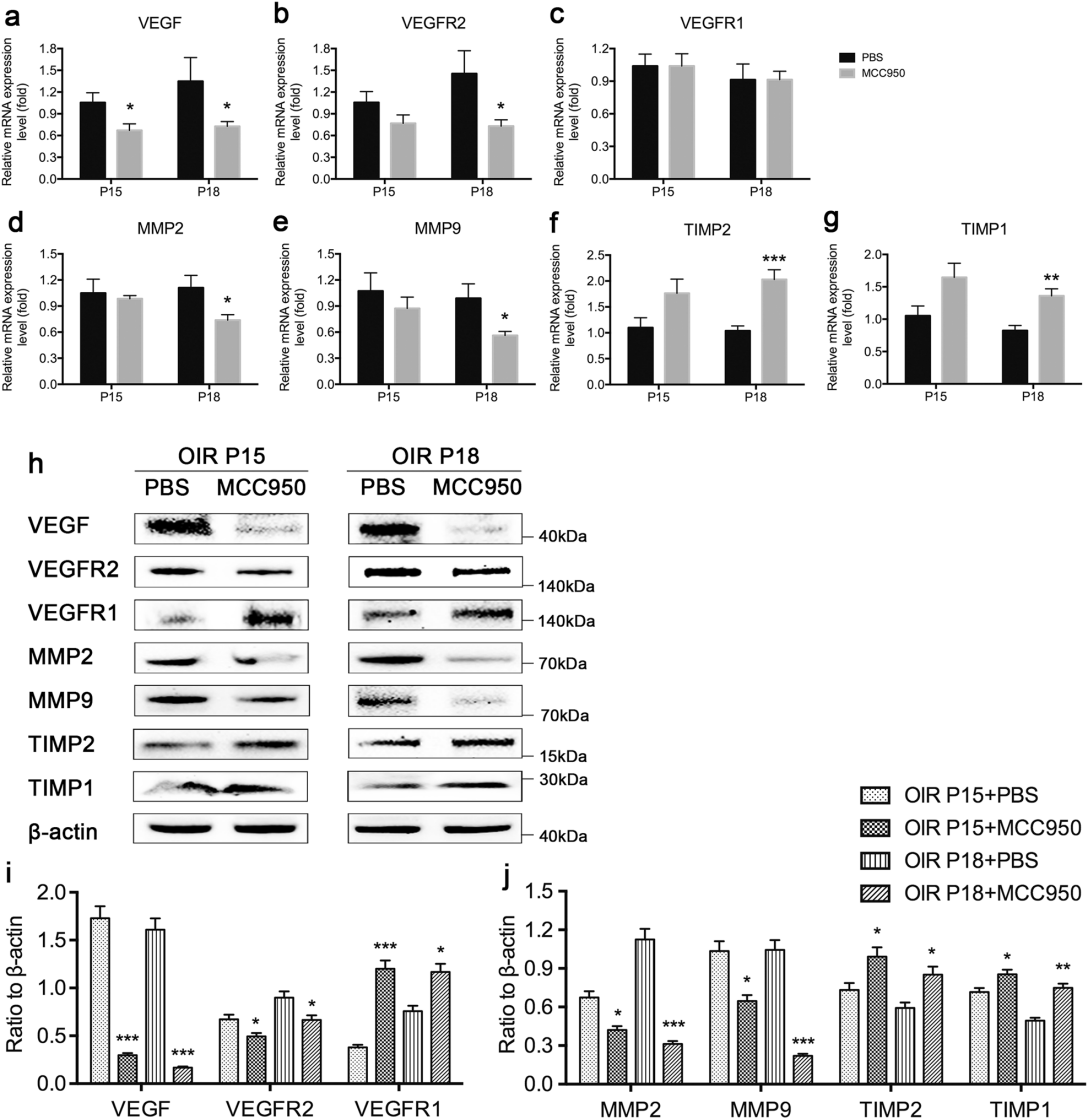
MCC950 regulated the expression of angiogenesis-associated molecules in OIR mice. **a**–**g** qRT-PCR was performed to quantify the mRNA expression of angiogenic and antiangiogenic genes, such as VEGF, VEGFR2, VEGFR1, MMP2, MMP9, TIMP2 and TIMP1, in the PBS and MCC950 groups (*n* = 6–8 mice/group). **h**–**j** Western blotting was performed to determine the protein expression levels of these factors (*n* = 3 mice/group). Data are presented as the mean ± SEM from three independent experiments and were analyzed by two-tailed Student’s *t* test. *P < 0.05, **P < 0.01, ***P < 0.001. OIR oxygen-induced ischemic retinopathy mice, P postnatal day.

qRT-PCR and western blot assays showed that angiogenesis-related factors, such as VEGF, VEGF receptor (VEGFR)2, matrix metalloproteinase (MMP)2, and MMP9, significantly decreased while antiangiogenic factors, such as VEGFR1, tissue inhibitor of metalloproteinases (TIMP)1 and TIMP2, significantly increased at the mRNA and protein levels at P18 (with the exception of VEGFR1 mRNA) (Fig. 5).

### Intravitreal injection of MCC950 increased the density and function of pericytes

**Fig. 6:**
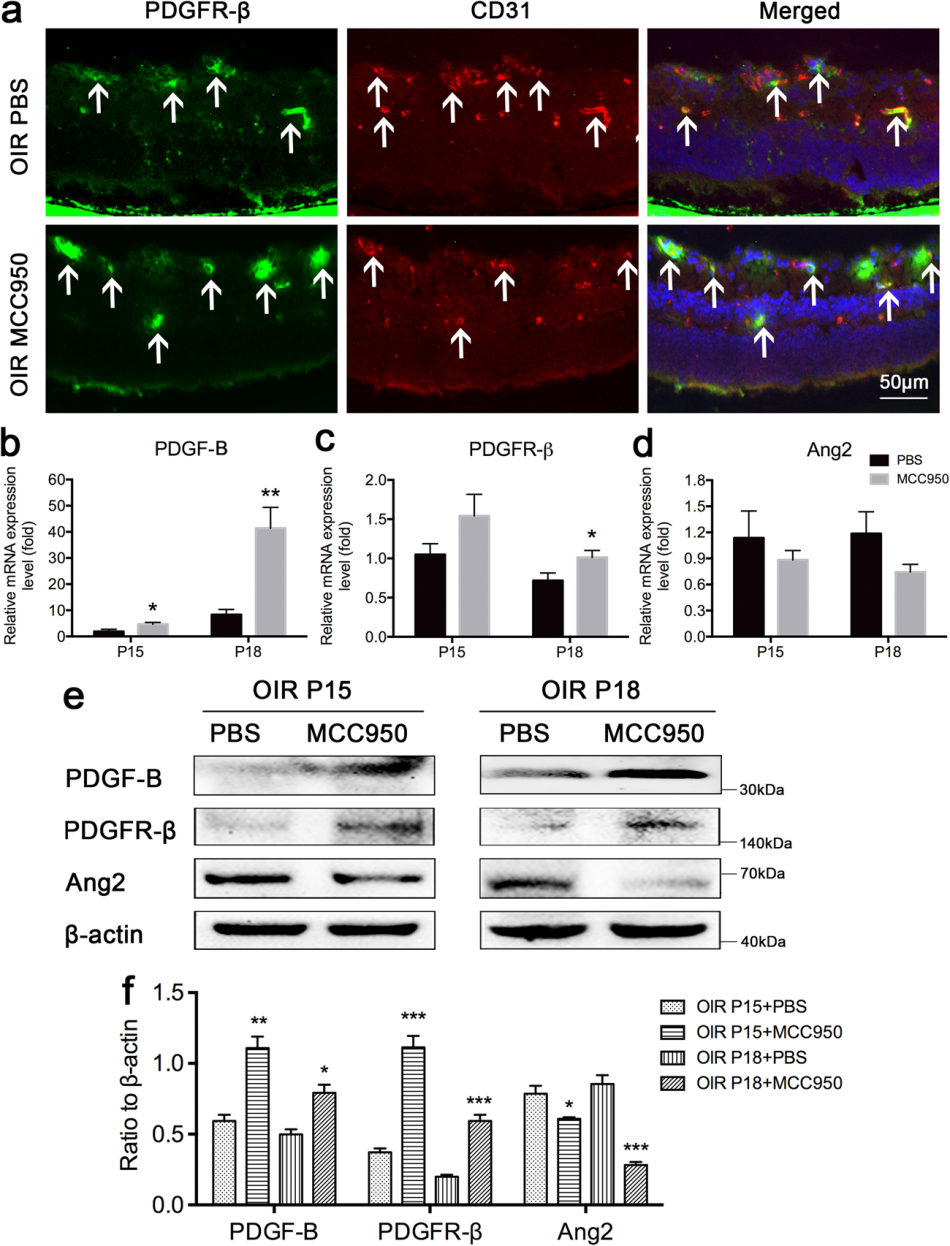
MCC950 regulated the density of pericytes and the expression of pericyte-related molecules. **a** Immunofluorescence staining for PDGFR-*β* (a marker of pericytes) and CD31 (a marker of endothelial cells) in PBS-treated and MCC950-treated OIR mice at P18 (*n* = 3 mice/group). Arrowheads point to positive areas. **b**–**d** The mRNA levels of pericyte-related genes, such as PDGF-B, PDGFR-*β*, and Ang2, in PBS-treated and MCC950-treated OIR mice were detected by qRT-PCR (*n* = 6–8 mice/group). **e**, **f** Protein levels of PDGF-B, PDGFR-*β* and Ang2 were detected by western blotting (*n* = 3 mice/group). Data are presented as the mean ± SEM from three independent experiments and were analyzed by two-tailed Student’s *t* test. *P < 0.05, **P < 0.01, ***P < 0.001. OIR oxygen-induced ischemic retinopathy mice, P postnatal day.

Immunofluorescence staining showed that compared with PBS-treated OIR mice, in MCC950-treated OIR mice, RNV decreased while the number of platelet-derived growth factor receptor-*β* (PDGFR-*β*)-labeled pericytes around RNV increased significantly (Fig. 6a). Retinal mRNA levels of platelet-derived growth factor-B (PDGF-B) and PDGFR-*β* were significantly increased, while those of angiopoietin2 (Ang2) were decreased in MCC950-treated OIR mice compared with PBS-treated mice (Fig. 6b–d). The same result was found in western blot assays (Fig. 6e–f). All the results above indicated that MCC950 could be used to treat RNV retinopathy by enhancing the density and function of pericytes and regulating the expression of endothelial cell function-related molecules.

### rIL-1*β* and rIL-18 affected the expression of angiogenesis-associated and pericyte function-associated molecules and the proliferation and migration of human retinal microvascular endothelial cells (HRMVECs) and human retinal microvascular pericytes (HRMVPCs)

**Fig. 7:**
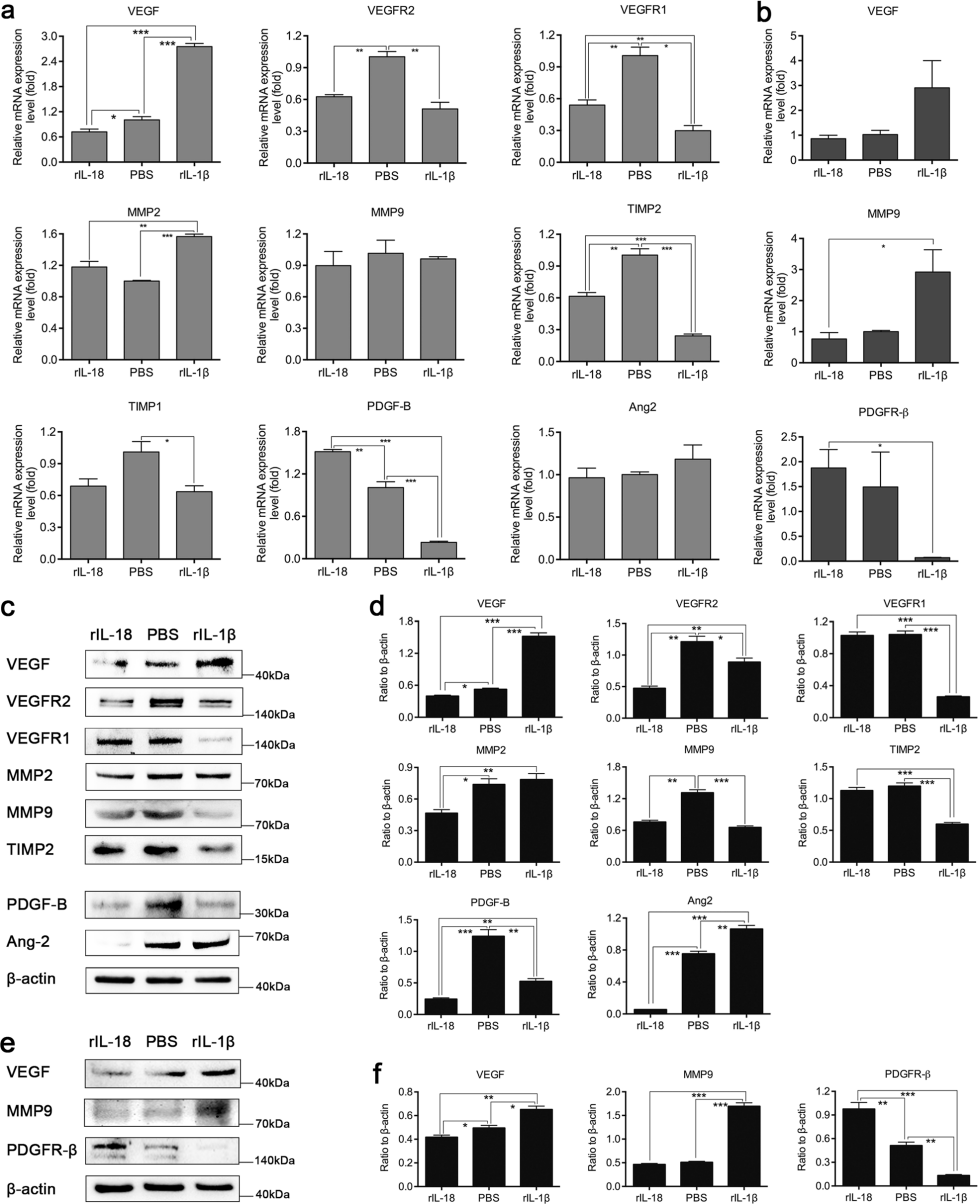
Effect of rIL-1*β* and rIL-18 on the expression of angiogenesis-associated and pericyte-related molecules in HRMVECs and HRMVPCs. qRT-PCR analysis of the mRNA expression of angiogenesis-associated molecules such as VEGF, VEGFR2, VEGFR1, MMP2, MMP9, TIMP2, and TIMP1 and pericyte-related molecules such as PDGF-B and Ang2 in HRMVECs (**a**) treated with PBS, rIL-18 or rIL-1*β* and VEGF, MMP9, and PDGFR-*β* in HRMVPCs (**b**) treated with PBS, rIL-18 or rIL-1*β* (*n* = 3 wells/group). Western blot analysis of the expression of the above mentioned molecular proteins in HRMVECs (**c**, **d**) and HRMVPCs (**e**, **f**) (*n* = 3 wells/group). Data are presented as the mean ± SEM from three independent experiments and analyzed by two-tailed Student’s *t* test. *P < 0.05, **P < 0.01, ***P < 0.001.

**Fig. 8:**
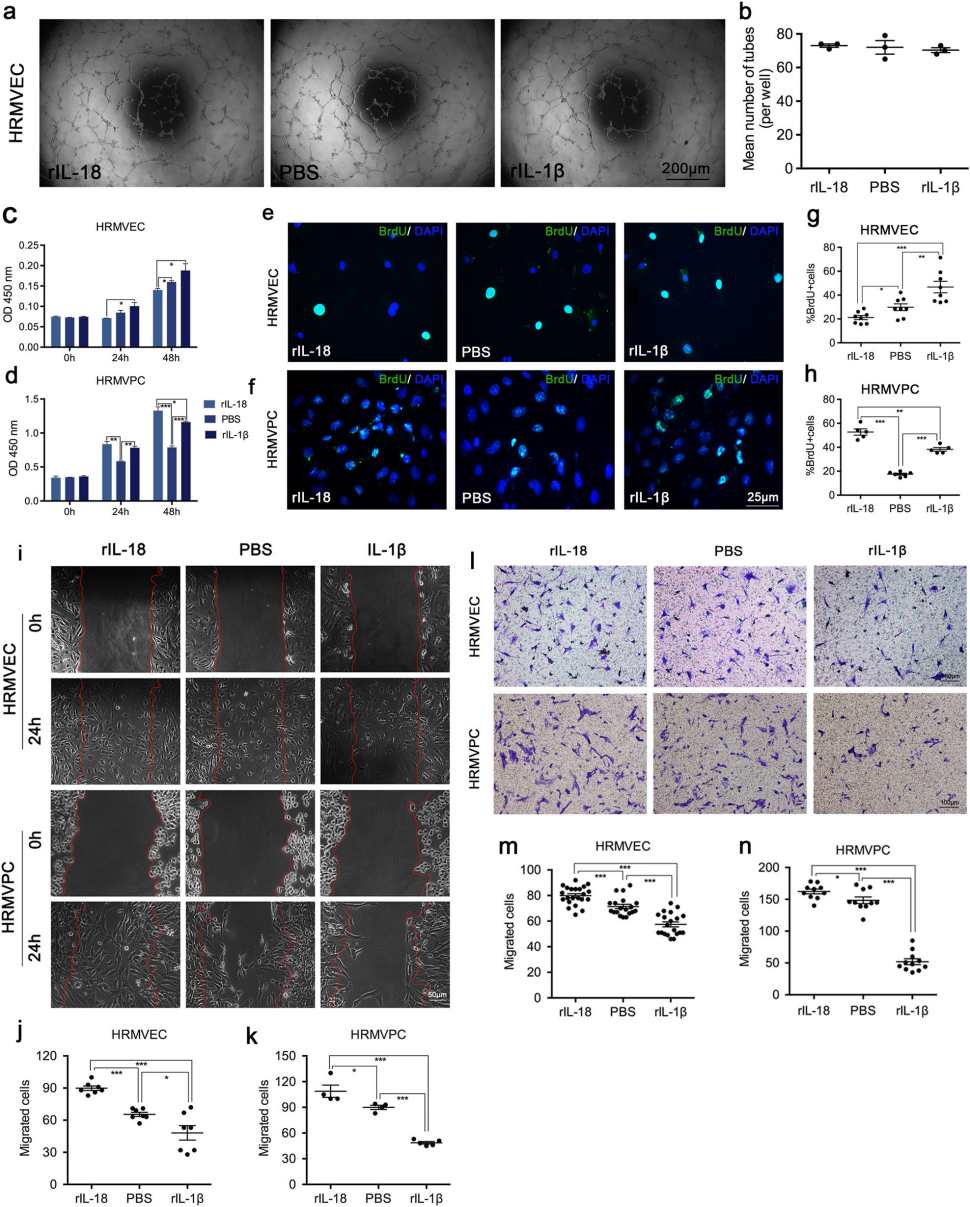
Effect of rIL-1*β* and rIL-18 on tube formation, cell proliferation, and migration in HRMVECs and HRMVPCs. **a**, **b** The average number of tube-like structures in HRMVECs for each field was statistically analyzed in the PBS, rIL-18, and rIL-1*β* groups (*n* = 3 wells/group). **c**–**h** CCK8 assay and BrdU labeling assay were performed to detect the proliferation of HRMVECs and HRMVPCs treated with PBS, rIL-18, or rIL-1*β*. The ratio of BrdU-positive cells was calculated in five to eight different regions. **i**–**k** A wound-healing migration assay was used to measure the horizontal migration of HRMVECs (*n* = 7 regions/group) and HRMVPCs (*n* = 4–5 regions/group). **l**–**n** Transwell assays were used to detect the vertical migration of HRMVECs (*n* = 19–22 regions/group) and HRMVPCs (*n* = 9–11 regions/group). Data are presented as the mean ± SEM from three independent experiments and were analyzed by two-tailed Student’s *t* test. *P < 0.05, **P < 0.01, ***P < 0.001.

In order to detect the direct role of IL-1*β* and IL-18 on the molecules expressed in HRMVECs and HRMVPCs, we performed qRT-PCR and western blotting assays with rIL-1*β* or rIL-18 treated cells and the results were as follows: compared to the rIL-1*β* treated group, the rIL-18 treated group showed significantly decreased expression of VEGF, VEGFR2, MMP2, and Ang2 in HRMVECs and VEGF and MMP9 in HRMVPCs, while the expression of VEGFR1, TIMP2, and PDGF-B in HRMVECs and PDGFR-*β* in HRMVPCs significantly increased at the mRNA or protein level (Fig. 7). We also wanted to know whether IL-1*β* and IL-18 could have a direct impact on tube formation, cell proliferation, and migration. The tube formation assay (Fig. 8a) results showed that the endothelial tube numbers in the three groups, the rIL-18 group, PBS group, and rIL-1*β* group, were not significantly different (Fig. 8b, *P* > 0.05), which means that rIL-18 and rIL-1*β* have no direct effect on the formation of NV. CCK8 assays showed that compared with the PBS treatment, rIL-18 played an inhibitory role, while rIL-1*β* played a facilitative role in the proliferation of HRMVECs (Fig. 8c). Both rIL-18 and rIL-1*β* can promote the proliferation of HRMVPCs, but the effect of rIL-18 on the proliferation of HRMVPCs was stronger than that of rIL-1*β* (Fig. 8d). Similar experimental results were obtained by BrdU labeling assay (Fig. 8e–h). Wound-healing migration assays and Transwell assays showed that rIL-18 promoted HRMVEC and HRMVPC migration, while rIL-1*β* suppressed HRMVPC and HRMVPC migration (Fig. 8i–n).

## Discussion

RNV is associated with vision loss in a number of ocular diseases. Activation of the immune system is thought to be related to the progression of RNV-associated diseases, but the pathway remains to be determined. The role of the NLRP3 inflammasome has been well studied in several diseases, but its role in ocular NV is conflicting. Some studies have suggested that the dysregulation and activation of the NLRP3 inflammasome may damage the immune response of the retina, leading to loss of the blood–retinal barrier, vascular leakage and the formation of DR^15^. Others have indicated that NLRP3 mediates the increase in IL-18 concentration, thus preventing the progression of choroidal neovascularization in wet AMD^11^. The aim of this study was to determine the role of the NLRP3 inflammasome in RNV and to explore the underlying mechanisms in OIR mice. In our experiment, we observed that the expression of NLRP3 and CAS1 began to increase at early stage of RNV formation (before P15). At later stage of RNV formation, the expression of NLRP3 and CAS1 significantly increased, while at the stage of RNV regression, it showed a relatively low expression level of NLRP3 and CAS1. This suggested that NLRP3 inflammasome participated in the whole process of RNV formation, which was consistent with previous research results^12^ while the complex regulatory mechanisms need to be studied in our further experiment. We also found that the expression of pro-CAS1 and activated CAS1 was inconsistent at different time points (for example, at P21). This variance may be due to the fact that pro-CAS1 was cleaved into activated CAS1 and tended to normal level, while the activated CAS1 did not degrade and maintained at a high level in the retinas of OIR mice at P21. We have observed the NLRP3 inflammasome activation in the retinas of OIR model mice, but have not yet determined whether it is pathogenic or protective. In previous studies, IL-1*β* and IL-18 were considered downstream action sites of caspase-1^11^. Over time, the expression of pro-IL-1*β* increased gradually and peaked at P21 in the retinas of OIR mice. We also observed that the expression levels of activated IL-1*β* and activated CAS1 at various time points were not consistent and this may be related to a number of factors including, but not limited to, the expression level of pro-IL-1*β*, the functional efficiency of cleaved CAS1 and the degradation rate of activated IL-1*β*, in addition to other complex regulatory mechanisms in biological individuals. The specific mechanisms will be further studied in ongoing research. Previous studies have reported low doses (5 μg/ml) of IL-1*β* conferred photoreceptor rescue and retinal protection in the inherited retinal degeneration rats^16–18^, after intravitreal injection of IL-1*β*, the number of acellular capillaries has tripled^19^. Researchers also observed that the IL-18 expression was decreased in the OIR model mice^20^ and the expression of VEGF and bFGF was upregulated, vasodilation and vascular leakage in the retinas of IL-18^−/−^ mice^20,21^. Some studies have shown that IL-18 inhibits choroidal neovascularization and may be as a potential treatment for wet AMD^22^. Interestingly enough, we observed that the expression and activation of IL-1*β* and IL-18 exhibited the opposite pattern in the retinas of OIR mice. We also found that when the activation of CAS1 was at low level, both the activation levels of IL-1*β* and IL-18 increased, while following the increase of CAS1 activation, the IL-1*β* activation level increased and the IL-18 activation level was inhibited. We speculated that it might be because of at the early stage of RNV formation, a small amount of IL-18 and IL-1*β* activation levels in the retina played a protective role. With the activation of CAS-1 and the expression of IL-1*β* increased in the later stage, the activation level of IL-1*β* increased significantly while the activation level of IL-18 inhibited, which aggravated the inflammatory response of retina. As a specific inhibitor of NLRP3, the protective effect of MCC950 on pulmonary inflammation, kidney fibrosis and other diseases has been well described^23–27^, but its role in OIR mice is unknown. Therefore, we studied the effect of MCC950 on the NLRP3 inflammasome in OIR mice and found that MCC950 significantly inhibited the activity of CAS1 and reversed the activation pattern of IL-1*β* and IL-18. We also used THP-1 cells as a supplementary experiment and found that under hypoxia, the NLRP3 inflammasome and IL-1*β* were significantly activated, while IL-18 activation was significantly decreased. After MCC950 intervention, CAS1 activation decreased, and IL-1*β* and IL-18 activation was reversed, which was consistent with our experimental results in vivo. In previous studies, the researchers thought that NLRP3 prevented the progression of choroidal neovascularization by increasing the IL-18 concentration^11^ while in the study of alcohol-induced liver injury, other researchers found the loss of the NLRP3 inflammasome reduced the amount of active IL-1*β* but dramatically increased the amount of active IL-18 in NLRP3^−/−^ mice^28^. This means that the exact relationship between IL-18 and NLRP3 inflammasome is not clear. Our results showed that the production of activated IL-18 was not consistent with CAS-1 activation while the precise mechanisms remained unclear and the possible reasons were as follows: in our experiment, we found that when CAS1 was in a low activation state (for example, at OIR P13), IL-1*β* and IL-18 were activated, especially IL-18, which indicated that other factors may be involved in the activation of IL-1*β* and IL-18. Previous studies have shown that CAS-1 activation was not the only way to activate pro-IL-1*β* and pro-IL-18^29^. Enzymes other than CAS-1, such as metalloproteinases (Meprin α or Meprin *β*) and macrophage- and neutrophil-derived neutral serine proteinases (protease 3), can also cleave pro-IL-1*β* to obtain active IL-1*β*^30,31^. Similar to CAS-1-independent processing of pro-IL-1*β*, epithelial cell-derived metalloproteinase Meprin *β*, NK cell-derived granzyme B, and mast cell-derived chymase can also cleave pro-IL-18 to obtain active IL-18^32^. Both IL-1*β* and IL-18 are members of the IL-1 family^29^, the activation of IL-18 and IL-1*β* may play a competitive role in the OIR mouse model; Different from the expression levels of IL-1*β* and IL-18 in other research models, the expression levels of IL-1*β* and IL-18 were opposite in our OIR mouse model (higher IL-1*β* and lower IL-18). We speculated whether the activation level of IL-1*β* and IL-18 was related to the expression level of IL-1*β* and IL-18, and CAS1 may has certain selectivity to the activation of different IL-18 and IL-1*β* expression levels; we also speculated that CAS1 with different activation degree may have certain selectivity to the activation of IL-18 and IL-1*β* under different environments. However, the above reasons are only speculation, and its specific mechanism will be further studied in our future work. We also wanted to evaluate the effect of MCC950 on RNV in the OIR model mice, so the following series of studies were carried out. We found that MCC950 significantly decreased the number of acellular capillaries, inhibited the formation and leakage of RNV. We speculated that MCC950 might reverse the activation patterns of IL-1*β* and IL-18 and lead to the above results, so further experiments were performed.

Previous studies have indicated that VEGF-A binds and activates receptor tyrosine kinase VEGFR2 on endothelial cells, triggering vascular growth and endothelial cell proliferation, migration, survival and increasing vascular permeability^27,33–35^. The more VEGF molecules interact with VEGFR2, the stronger the promoting effect on blood vessels^36^. Mice lacking VEGFR1 died of vascular overgrowth, and VEGFR1 usually regulates vascular growth by controlling the speed of endothelial cell division in vitro and in vivo^37^. TIMP-1 and TIMP-2 specifically inhibit the activity of MMP-9 and MMP-2, inhibit the division of endothelial cells and the degradation of extracellular matrix, and reduce angiogenesis^38^. By detecting the expression of these related factors, we found that the expression of VEGF, VEGFR2, MMP2, and MMP9, which promote angiogenesis, decreased, while the expression of VEGFR1, TIMP2, and TIMP1, which inhibit angiogenesis, increased after intervention with MCC950. These results suggest that MCC950 inhibits the formation of RNV by regulating the expression of angiogenesis-associated molecules.

It is known that the presence of pericytes in new capillary buds is an important step in angiogenesis. Although the function of pericytes is not completely clear at present, it is generally believed that they can help stabilize the blood vessel wall and prevent vascular leakage. During the development of blood vessels, PDGF-B released by endothelial cells activates the tyrosine kinase PDGFR-*β* on the surface of pericytes and recruits pericytes^39–41^. In PDGF-B or PDGFR-*β* knockout mice, the number of pericytes decreased significantly, and various vascular defects were observed, such as vascular proliferation, microvascular expansion and upregulation of VEGF-A expression^41–44^. All these studies suggest that PDGF-B and PDGFR-*β* have great effects on the development and maturation of blood vessels. Some studies have also indicated that the increase in Ang2 might be another factor in the loss of pericytes^45,46^. In our studies, we found that the number of pericytes and the expression of PDGF-B and PDGFR-*β* significantly increased, while Ang2 expression significantly decreased in the MCC950 group. We carried out in vitro experiments and found that compared to the rIL-1*β* group, the rIL-18 group displayed significantly decreased expression of VEGF, VEGFR2, MMP2 and Ang2 in HRMVECs and VEGF and MMP9 in HRMVPCs, while the expression of VEGFR1, TIMP2, and PDGF-B in HRMVECs and PDGFR-*β* in HRMVPCs significantly increased at the mRNA or protein level. This means that IL-1*β* and IL-18 played opposite roles in regulating the functions of endothelial cells and pericytes. The tube formation assay results demonstrated that rIL-18 and rIL-1*β* had no direct effect on the formation of NV. The cell proliferation assays showed that rIL-18 played an inhibitory role, while rIL-1*β* played a facilitative role, in the proliferation of HRMVECs. Both rIL-18 and rIL-1*β* could promote the proliferation of HRMVPCs, but the effect of rIL-18 on the proliferation of HRMVPCs was stronger than that of rIL-1*β*. The cell migration assay showed that rIL-18 promoted while rIL-1*β* suppressed HRMVEC and HRMVPC migration. These results indicated that IL-1*β* and IL-18 could participate in the formation of RNV by directly influencing the proliferation and migration of endothelial cells and pericytes rather than by directly influencing tube formation.

In summary, the present study demonstrated for the first time that inhibiting the NLRP3 inflammasome with MCC950 could regulate the function of endothelial cells and pericytes by reversing the IL-1*β*/IL-18 activation pattern to ameliorate RNV and leakage. This study could provide an experimental basis for the identification of new therapeutic targets of RNV-associated diseases.

## Materials and methods

### Animals and ethics statement

All C57BL/6 mice in this study were specific pathogen-free. All animal procedures were performed according to the Guide for the Care and Use of Laboratory Animals with the approval (SYXK-2011–0026) of the Scientific Investigation Board of Shanghai Jiao Tong University School of Medicine, Shanghai, China.

### Mouse model of oxygen-induced ischemic retinopathy (OIR)

The mouse model of OIR was established as previously described^47^. The OIR mice were randomly (random number method) divided into several groups: one eye was given an intravitreal injection of an inhibitor of NLRP3, MCC950 (1 μL, Selleck Chemicals, Houston, TX, USA) at different concentrations (1 μM, 10 μM, 100 μM, and 1 mM); the contralateral eye was treated with PBS (1 μL). According to the different experimental purposes, ocular samples were collected at different time points.

### Immunofluorescence staining and retinal flat-mount

Cross-sections were prepared and assayed as described previously^7^. Primary antibodies against the following antigens were used: NLRP3 (Cell Signaling Technology, MA, USA), NLRP3 (Invitrogen, CA, USA), ASC, CAS1 and PDGFR-*β* (Santa Cruz Biotechnology, TX, USA), IL-1*β* and IL-18 (Abcam, Cambridge, UK). After incubation with Alexa Fluor 555 donkey anti-rabbit secondary antibody (Invitrogen) plus fluorescein isothiocyanate (FITC)-*Griffonia simplicifolia* (GSA)-Lectin-B4 (Vector Laboratories, Inc., CA, USA), FITC anti-rabbit IgG antibody (Cell Signaling Technology) plus F4/80-PE (eBioscience, Inc., San Diego, CA, USA), FITC anti-rabbit IgG antibody plus anti-mouse CD31(PECAM-1)-PE (eBioscience), FITC anti-rat IgG antibody (Abcam) plus Alexa Fluor 555 donkey anti-rabbit secondary antibody (Invitrogen) for 1 h at room temperature in the dark, the sections were stained with 4′,6-diamidino-2-phenylindole (DAPI, Beyotime Biotechnology, Shanghai, China) for 5 min, and images were captured under a fluorescence microscope (Nikon, New York, USA). Retinas from OIR mice were carefully dissected, incubated with FITC-GSA-Lectin (Vector Laboratories), flat-mounted, and then imaged under a fluorescence microscope or confocal fluorescent microscopy (Carl Zeiss, Obercohen, Germany) at P18^7^.

### Periodic acid-schiff (PAS)/hematoxylin staining

Retinas fixed with 4% neutral formalin were incubated in 3% trypsin at 37 °C until the solution was turbid and the tissue appeared disintegrated. Retinas were shaken gently to separate the vascular network from attached retina tissue, and washed in PBS. A thin layer of translucent vascular network was observed under the microscope and then placed then on a glass slide, dried at room temperature, and subsequently stained with PAS/hematoxylin^48^. The histopathological changes of retinal vessels were photographed and the number of acellular capillaries analyzed.

### Evans blue assay

OIR mice were anesthetized at P18, Evans blue (45 mg/kg) was perfused through the left ventricle for at least 5 min, and mice were then sacrificed. After fixation, the whole retina was dissected, flat-mounted and imaged with a fluorescence microscope. Retinas from eyes were dried in a vacuum for 5 h, weighed, placed in a 1.5 mL centrifuge tube containing 200 μL of formamide (Sigma-Aldrich, St. Louis, MO, USA), incubated at 70 °C for 8 h, and then centrifuged at 120,000 rpm and 4 °C for 60 min. Totally, 70 μL of supernatant was collected, and the absorbance was measured under spectrophotometer at 620 and 720 nm. According to the standard curve of Evans blue in formamide, the dye concentration was calculated, and the content of Evans blue was standardized by the dry weight of the retina (ng/mg)^48^.

### Quantitative reverse transcription polymerase chain reaction (qRT-PCR)

Total RNA was isolated, and cDNA synthesis was performed using a cDNA synthesis kit (Roche, Basel, Switzerland). qRT-PCR was carried out using SYBR Green Mix (Roche) on the ABI 7500 Real-time PCR system (Applied Biosystems, CA, USA). CyclophilinA was used as an endogenous internal control and the 2^−ΔΔCt^ method was used to calculate the relative expression. The primers for NLRP3^49^, ASC^50^, CAS1^51^, IL-1*β*^7^, IL-18, VEGF, VEGFR2, VEGFR1, MMP2, MMP9^52^, TIMP1, TIMP2, PDGF-B, PDGFR-*β*, Ang2, cyclophilinA^7^, and GAPDH are listed in Supplementary Table 1.

### Western Blotting

Proteins of retinal samples and cells were isolated, and western blotting was performed as previously described^50^. The primary antibodies included antibodies against NLRP3, ASC, CAS1, IL-1*β*, IL-18, VEGF, VEGFR2, VEGFR1, MMP2, MMP9, TIMP1, TIMP2, PDGFR-*β*, PDGF-B, and Ang2. See Supplementary Table 2 for details. Protein band intensities were measured by ImageJ software (National Institutes of Health, MD, USA).

### Cell culture and tube formation assay

HRMVECs (cAP-0010, Angio-Proteomie, MA, USA) and HRMVPCs (cAP-0025, Angio-Proteomie) were cultured with endothelial cell medium (ECM; Sciencell, CA, USA) and Dulbecco’s modified Eagle’s medium (DMEM; Gibco, Grand Island, NY, USA) containing 10% fetal bovine serum (Gibco) and 100 U/mL penicillin–streptomycin, respectively, at 37 °C in 5% CO_2_. Cells in the logarithmic growth stage were inoculated into 6 cm culture dishes and adhered after 24 h, followed by stimulation with PBS, recombinant human IL-1*β* (rIL-1*β*, 100 pg/mL, R&D systems, Minneapolis, MN, USA) or rIL-18 (100 ng/mL, R&D systems) for qRT-PCR (12 h) and western blot (24 h). Totally, 50 μL of basement membrane matrix (BMM, BD Biosciences, San Jose, CA, USA) was added to 96-well plate and hardened at 37 °C for 30 min. HRMVEC (3 × 10^4^) suspensions with ECM containing PBS, rIL-1*β* or rIL-18 were plated onto the surface of BMM, incubated for tube formation at 37 °C for 9 h and examined using an inverted microscope. Endothelial tube numbers were counted as described previously^53^.

### Cell proliferation assay

HRMVECs and HRMVPCs at the logarithmic growth stage were inoculated into 96-well plates (5 × 10^3^ cell density/well). After 24 h, the original medium was replaced with medium containing PBS, rIL-1*β* or rIL-18, and the cells were incubated at 37 °C for 0, 24, and 48 h. After CCK-8 reagent was added for incubation at 37 °C for 2 h, the absorbance was measured with a microplate reader at 450 nm (Thermo Fisher Scientific. Inc., MA, USA). BrdU at a final concentration of 10 μM was added to HRMVEC and HRMVPC media containing PBS, rIL-18 or rIL-1*β* and incubated for 48 h. After fixation, denaturation, neutralization, blocking, incubation with BrdU primary antibody and secondary antibody, and nuclear staining, the cells were imaged, and the ratio of BrdU-positive cells was calculated in five to eight different regions.

### Cell migration assay

A wound-healing migration assay was used to measure the horizontal migration of HRMVECs and HRMVPCs. Briefly, cells cultured to the logarithmic growth stage were inoculated in 6-well plates (2.5 × 10^6^ cells/well), scratched with pipette tips after 24 h, and washed with PBS to remove the cell debris. The cells were cultured with medium containing PBS, rIL-1*β* or rIL-18 for 24 h and imaged by microscopy at 0 and 24 h. Transwell assays were used to detect the vertical migration of HRMVECs and HRMVPCs. Cells (4 × 10^4^ cells/well) were resuspended in 200 μL serum-free medium and added to the upper chamber of the Transwell insert (Corning, Tewksbury, MA, USA). Then, 500 μL of medium containing PBS, rIL-1*β* or rIL-18 was added into the lower chamber. After incubation for 24 h, the filter membranes were fixed with 4% paraformaldehyde and stained with 1% crystal violet. The nonmigrated cells that adhered to the upper chamber were wiped off with cotton swabs, and the cells on the lower surface were imaged by microscopy.

### Statistical analysis

Animals with poor physical condition were excluded before randomization, experimental intervention and data analysis. The samples were randomly divided into different groups by random number method. The experiments were repeated at least three times. In the process of data collection and analysis, the investigators were blinded to group allocation. Statistical analysis was performed by the two-tailed Student’s *t* test or Student–Newman–Keuls using SAS 9.0 software (SAS Institute Inc., Cary, NC, USA). Data are displayed as the mean ± S.E.M. A value of *P* < 0.05 was considered statistically significant.

## Supplementary information

Supplementary

Supplementary Figure S1

Supplementary Figure S2

Supplementary Figure S3

Supplementary Figure S4
