# Supplementary material for: Inhibiting the NLRP3 inflammasome with MCC950 ameliorates retinal neovascularization and leakage by reversing the IL-1β/IL-18 activation pattern in an oxygen-induced ischemic retinopathy mouse model
Source: Cell Death Dis. 2020 Oct 22;11(10):901. doi: 10.1038/s41419-020-03076-7 (PMC7582915; doi:10.1038/s41419-020-03076-7)
Supplement: Supplementary file 1 — Supplementary [file 41419_2020_3076_MOESM1_ESM.docx]

**Supplementary Information**

**Supplementary Materials and Methods**

**Cell Culture and Immunofluorescence Staining**

The human monocytic cell line (THP-1) cells (Cell Bank Chinese Academy of Science, Shanghai, China) were cultured with RPMI-1640 medium (Gibco) containing 10% fetal bovine serum (Gibco) and 100 U/mL penicillin-streptomycin at 37°C in 5% CO_2_. THP-1 cells at the logarithmic growth stage were differentiated into M0 macrophages with 100ng/mL phorbol-12-myristate-13-acetate (PMA) (Sigma-Aldrich) for 24 hours. The cells were randomly divided into three groups: normoxia group, hypoxia group, and MCC950 plus hypoxia group. The cells were fixed, permeabilized, blocked and incubated with primary antibodies against NLRP3 (Invitrogen) plus ASC (Santa Cruz Biotechnology), CAS1 (Santa Cruz Biotechnology), IL-1β and IL-18 (Abcam) at 4°C overnight and then incubated with the corresponding secondary antibodies: Alexa Fluor 555 donkey anti-rabbit secondary antibody (Invitrogen) plus FITC anti-rat secondary antibody (Abcam), FITC anti-rabbit secondary antibody (Invitrogen) at room temperature for 1 hour. After staining with DAPI (Beyotime Biotechnology), images were captured under a confocal fluorescent microscopy (Carl Zeiss).

**THP-1 cells western blot assays**

THP-1 cells at the logarithmic growth stage were inoculated into 6 cm culture dishes and were differentiated into M0 macrophages with 100ng/mL PMA (Sigma-Aldrich) for 24 hours. The M0 macrophages were randomly divided into two groups: one group was cultured under hypoxia condition (hypoxia incubator chamber: 1% 0_2_, 5% CO_2_, 94% N_2_) for 0 hours, 6 hours and 12 hours; the other group was pre-treated with different concentrations MCC950 (0nM, 1nM, 10nM, 100nM) for 6 hours under normoxic conditions and then cultured under hypoxic conditions for 12 hours. All cells were collected for western blot assay.

**Supplementary Figure Legends**

**Supplementary Figure S1 Immunofluorescence staining for F4/80 and ASC (or CAS1) in the retinas of normal and OIR mice.** The eyes from normal and OIR mice at P18 were cross sectioned and then stained for F4/80 and ASC, F4/80 and CAS1. Arrowheads point to positive areas.

**Supplementary Figure S2 Immunofluorescence staining for NLRP3 inflammasome, IL-1β and IL-18 in THP-1 cells.** Immunofluorescence staining of NLRP3, ASC, CAS1, IL-1β and IL-18 in normoxia-induced THP-1cells, hypoxia-induced THP-1cells, pre-treated with MCC950 and hypoxia-induced THP-1cells. “NLRP3+ASC” images demonstrate the colocalization of NLRP3 and ASC. Magnified z-stacks of confocal images clearly demonstrate the formation of ASC specks and the colocalization of NLRP3 and ASC.

**Supplementary Figure S3 NLRP3 inflammasome and IL-1β and IL-18 expression and activity detected in THP-1 cells under normoxia and hypoxia conditions by western blot.** (a) Western blot analysis of the protein expression of NLRP3, ASC, pro-CAS1, CAS1, pro-IL-1β, IL-1β, pro-IL-18 and IL-18. (b-i) Data were shown as mean ± SEM from three independent experiments and analyzed by Student-Newman-Keuls. **P < 0.05, **P < 0.01*.

**Supplementary Figure S4 NLRP3 inflammasome and IL-1β and IL-18 expression and activity detected in THP-1 cells pre-treated with MCC950 under hypoxic conditions by western blot.** (a) Western blot analysis of the protein expression of NLRP3, ASC, pro-CAS1, CAS1, pro-IL-1β, IL-1β, pro-IL-18 and IL-18 at different concentrations of MCC950 (0nM, 1nM, 10nM, 100nM) pre-treated hypoxia-induced THP-1 cells. (b-i) Data were shown as mean ± SEM from three independent experiments and analyzed by Student-Newman-Keuls. **P < 0.05, **P < 0.01, ***P < 0.001*.

**Supplementary Table 1: Primers for qRT-PCR**

| **species** | **Gene** | **forward sequence** | **Reverse sequence** |
| --- | --- | --- | --- |
| mouse | NLRP3 | TCCTGGTGACTTTGTATATGCGT | TTCT CGGGCGGGTAATCTTC |
|  | ASC | GCTGAGCAGCTGCAAACGA | ACTTCTGTGACCCTGGCAATGA |
|  | CAS1 | TGCCTGGTCTTGTGACTTGGA | CCTATCAGCAGTGGGCATCTGTA |
|  | IL-1β | TGCCACCTTTTGACAGTGATG | AAGGTCCACGGGAAAGACAC |
|  | IL-18 | CAGCAGTCCAACTGCAGACT | TGTGCCTGGATGCTTGTAAAC |
|  | VEGF | CACTTCCAGAAACACGACAAAC | TGGAACCGGCATCTTTATCTC |
|  | VEGFR2 | GGCGAATCACTCACACCAGT | GCAATTCTGTCACCCAGGGAT |
|  | VEGFR1 | GCAGTCTGAGAGGAGCTAAAG | GGAGAGGGAGCTTGCATAAA |
|  | MMP2 | GCTCTGTCCTCCTCTGTAGTTA | GGTACAGTCAGCACCTTTCTT |
|  | MMP9 | TGCACTGGGCTTAGATCATTC | TGCCGTCTATGTCGTCTTTATTC |
|  | TIMP1 | AGTGATTTCCCCGCCAACTC | TCCTTTTAGCATCCAGGTCCG |
|  | TIMP2 | TTCCTCTCCCCTGTCTCTACAC | TGTGCAAAAGAGGGAGTGCT |
|  | PDGF-B | TCAAGCTCGGGTGACCATTC | AAGGCTCCTGCACACTTGTT |
|  | PDGFR-β | CAAGAGAACTCTGCCTCGGG | CACCAGGTCCTTGCCTGATT |
|  | Ang2 | CACCACTTGCACACACAAAG | TTCTCACAGCCAATAA |
|  | cyclophilinA | CAGACGCCACTGTCGCTTT | TGTCTTTGGAACTTTGTCTGCAA |
| human | VEGF | CTTCAAGCCATCCTGTGTGC | CGCTCCAGGACTTATACCG |
|  | VEGFR1 | TAGTGTTGTGGGCTCTGTATTC | AGCTTCCTCAGCACACTATTT |
|  | VEGFR2 | AACCAAGGTACTTCGCAGGG | AACCAAGGTACTTCGCAGGG |
|  | MMP2 | GACACATCTGGGCAGTTGCTAA | GGTCACATCGCTCCAGACTT |
|  | MMP9 | GGTGATTGACGACGCCTTTG | GGACCACAACTCGTCATCGT |
|  | TIMP1 | TCGTCATCAGGGCCAAGTTC | TCCACAAGCAATGAGTGCCA |
|  | TIMP2 | CAGCTTTGCTTTATCCGGGC | ATGCTTAGCTGGCGTCACAT |
|  | PDGF-B | TGACTGAGCAGGAATGGTGAGA | CTCCTTCTTCCACGAGCCAA |
|  | PDGFR-β | TGCTCAGCAGAGTGTCATCC | GGTGCGGTTGTCTTTGAACC |
|  | Ang2 | TTGGCCGCAGCCTATAACAA | CCGCTGTTTGGTTCAACAGG |
|  | GAPDH | GGGAAACTGTGGCGTGAT | GAGTGGGTGTCGCTGTTGA |

**Supplementary Table 2: Antibodies used for western blot**

|  | **Cat.No.** | **WB** | **Source** |
| --- | --- | --- | --- |
| NLRP3 | 15101 | 1:1000 | Cell Signaling Technology |
| ASC | SC-22514-R | 1:500 | Santa Cruz Biotechnology |
| CAS1 | SC-56036 | 1:500 | Santa Cruz Biotechnology |
| IL-1β | ab9722 | 1:1000 | Abcam |
| IL-18 | ab71495 | 1:250 | Abcam |
| VEGF | 07-1420 | 1:500 | Millipore |
| VEGFR1 | SC-316 | 1:500 | Santa Cruz Biotechnology |
| VEGFR2 | 9698S | 1:1000 | Cell Signaling Technology |
| MMP2 | ab80737 | 1:1000 | Abcam |
| MMP9 | Ab38898 | 1:1000 | Abcam |
| TIMP1 | SC-5538 | 1:500 | Santa Cruz Biotechnology |
| TIMP2 | MAB13446 | 1:500 | Millipore |
| PDGFR-β | SC-374573 | 1:500 | Santa Cruz Biotechnology |
| PDGF-B | SC-7878 | 1:500 | Santa Cruz Biotechnology |
| Ang2 | ab8452 | 1:500 | Abcam |
| β-actin | E021020 | 1:1000 | EarthOx |
