# Supplementary figures and images for: Inhibiting the NLRP3 inflammasome with MCC950 ameliorates retinal neovascularization and leakage by reversing the IL-1β/IL-18 activation pattern in an oxygen-induced ischemic retinopathy mouse model
Source: Cell Death Dis. 2020 Oct 22;11(10):901. doi: 10.1038/s41419-020-03076-7 (PMC7582915; doi:10.1038/s41419-020-03076-7)

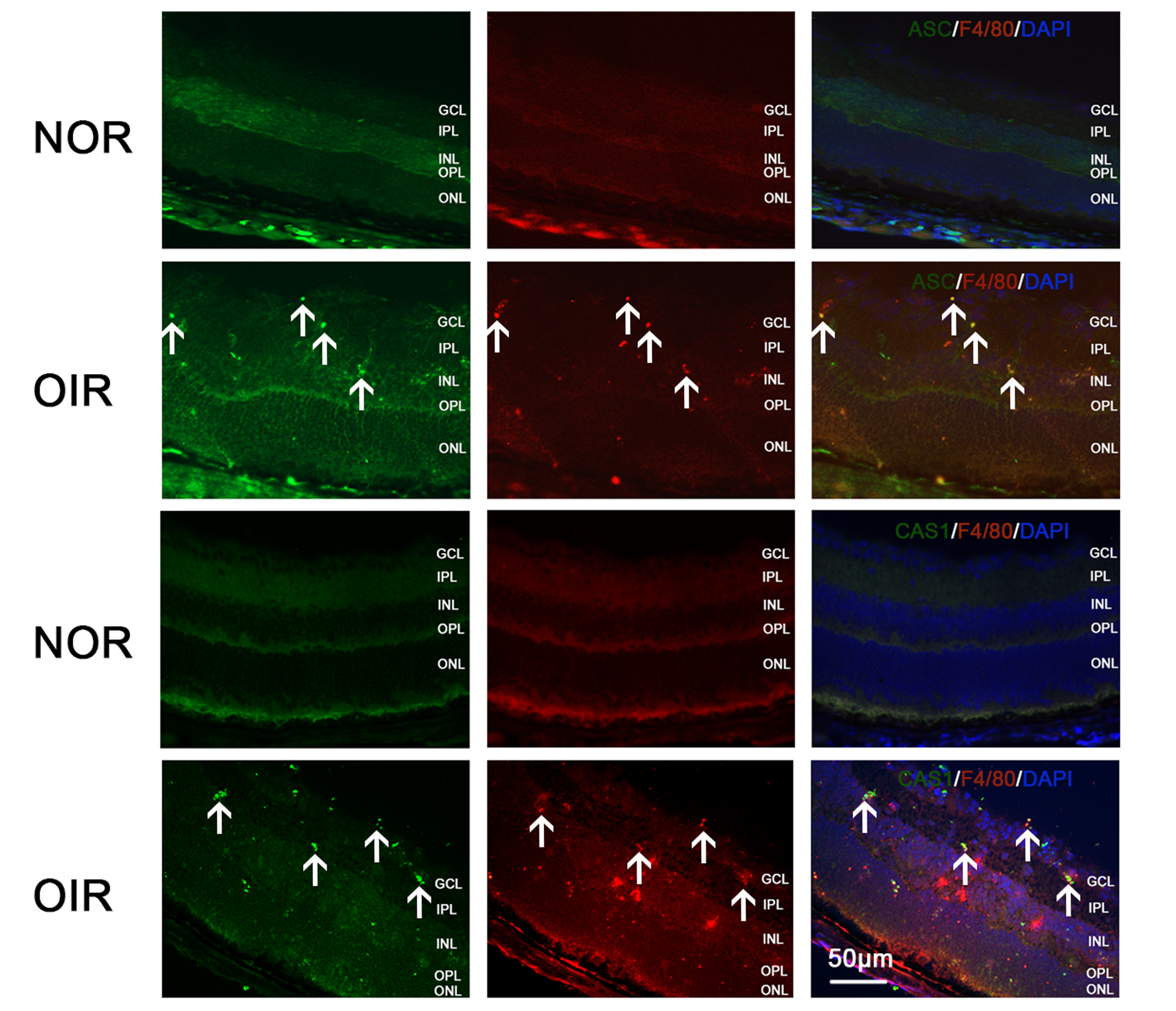

Supplement: Supplementary file 2 — Supplementary Figure S1 [file 41419_2020_3076_MOESM2_ESM.tif]

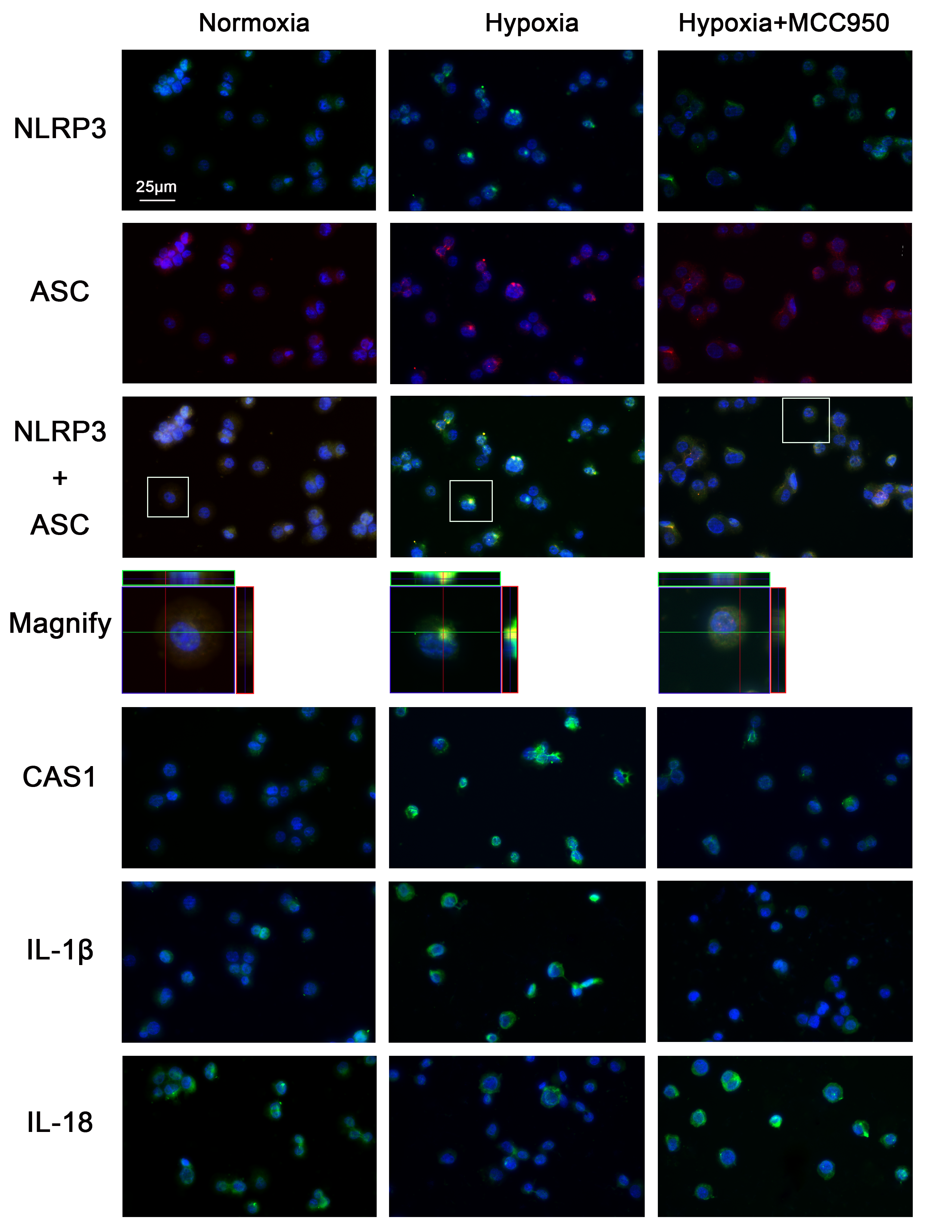

Supplement: Supplementary file 3 — Supplementary Figure S2 [file 41419_2020_3076_MOESM3_ESM.tif]

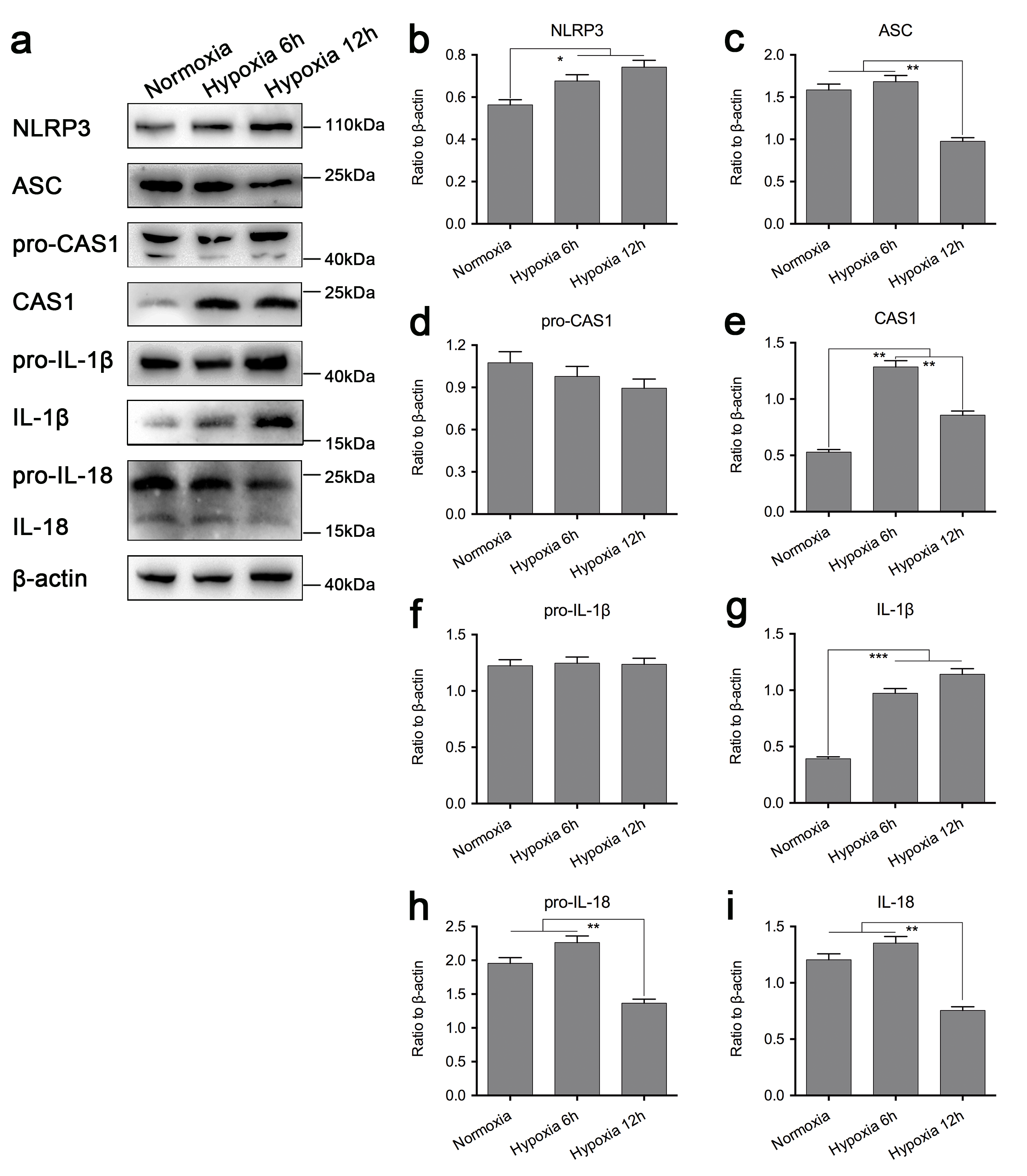

Supplement: Supplementary file 4 — Supplementary Figure S3 [file 41419_2020_3076_MOESM4_ESM.tif]

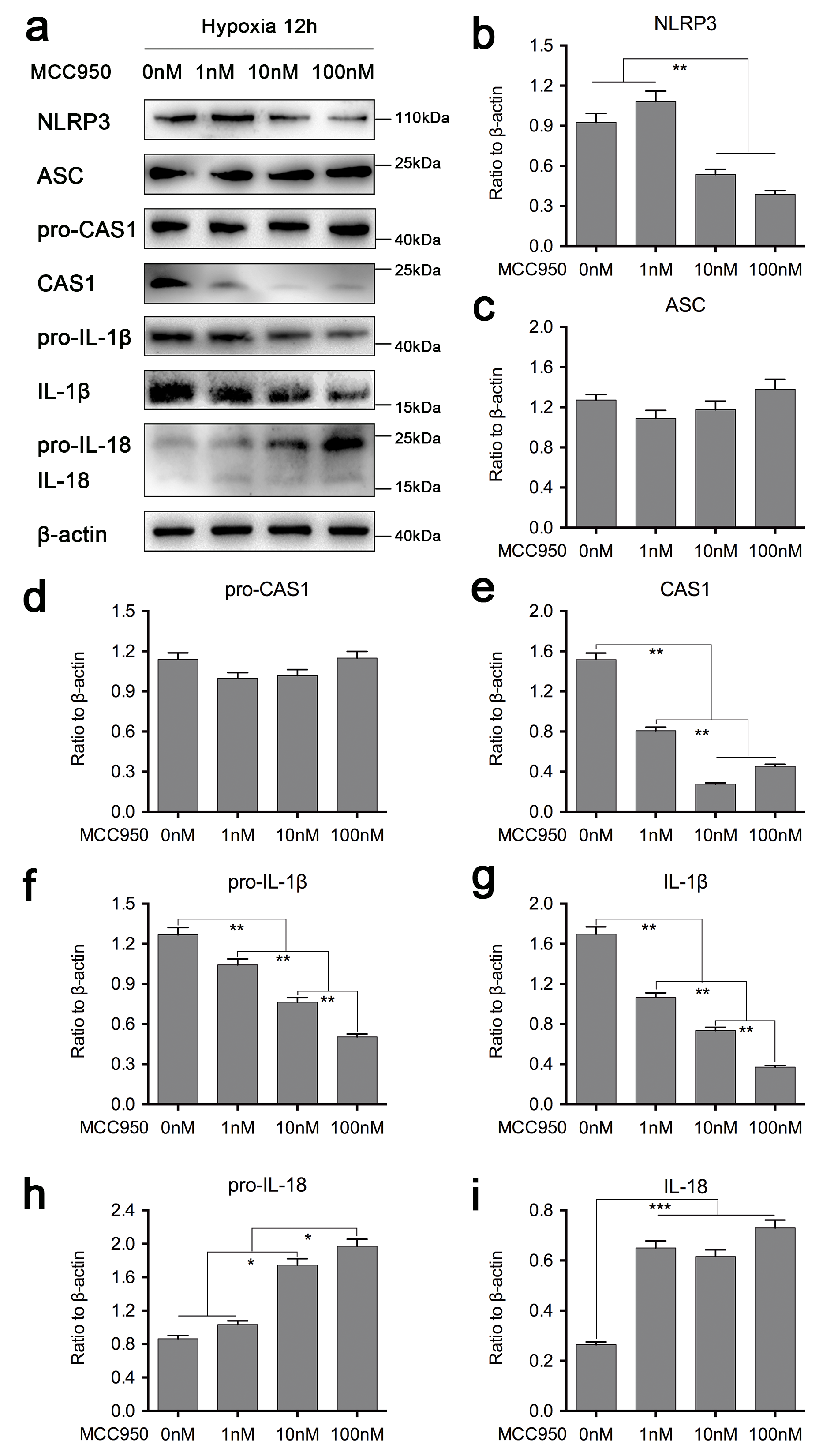

Supplement: Supplementary file 5 — Supplementary Figure S4 [file 41419_2020_3076_MOESM5_ESM.tif]
